# Social organization and habitat use shape the gut microbiome of a marine fish

**DOI:** 10.1111/1365-2656.70286

**Published:** 2026-06-10

**Authors:** Aina Pons, Eneko Aspillaga, Ignacio A. Catalán, Tomeu Viver, Robert Arlinghaus, Martina Martorell‐Barceló, Margarida Barcelo‐Serra, Josep Alós

**Affiliations:** ^1^ Mediterranean Institute for Advanced Studies (IMEDEA CSIC‐UIB) Esporles Balearic Islands Spain; ^2^ Department of Fish Biology, Fisheries and Aquaculture Leibniz Institute of Freshwater Ecology and Inland Fisheries Berlin Germany; ^3^ Division of Integrative Fisheries Management, Faculty of Life Sciences Humboldt‐Universität zu Berlin Berlin Germany; ^4^ Institute of Marine Research Tromsø Norway

**Keywords:** 16S, habitat, marine fish, microbiome, pearly razorfish, social network, space use

## Abstract

1. The gut microbiome hosts diverse bacterial communities that significantly influence individual spatial behaviour in animal societies. However, this relationship remains understudied in marine fish due to the challenges associated with measuring behavioural traits in free‐living fish and simultaneously obtaining gut microbiome composition data.
2. In this study, we conducted a field experiment to explore the relationship between space and habitat use, social organization, and gut microbiome composition in marine fish. We used a novel high‐resolution acoustic telemetry system to collect 7930 one‐day‐long movement trajectories from 232 individuals of *Xyrichtys novacula* of the same population (153 females, 79 males) near the coastline of Mallorca, Spain.
3. A subset of these individuals was recaptured to analyse the diversity of core and non‐core gut microbiome, quantified using operational phylogenetic units (OPUs) based on 16S rRNA gene amplicons through Illumina sequencing.
4. Individuals closer to *Posidonia oceanica* seagrass meadow had higher non‐core microbiome diversity, especially larger individuals. Multivariate analysis showed no significant differences in microbiome composition across the tested variables (i.e. body size, territory size, degree, strength, distance to the seagrass meadow, and sex), but males showed a visually greater, but non‐significant, variability in core microbiome composition. Core microbiome composition was weakly associated with social harem structure.
5. These findings indicate that gut microbiome composition is primarily shaped by local habitat conditions, while social organization may contribute weakly and indirectly, pending further experimental validation.

## Graphical abstract

This study provides the first evidence linking habitat use—and to a lesser extent social organization—to gut microbiome composition in a wild marine fish. The results indicate that local habitat conditions are the primary driver of microbial variation, while social effects are detectable but weak. These findings refine our understanding of the ecological determinants of host–microbiome relationships in the wild, highlighting habitat‐mediated processes as a dominant mechanism shaping microbial communities in natural populations.

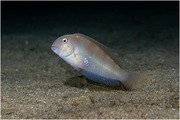

## 1 INTRODUCTION

The term gut microbiome refers to the collection of bacteria and their genes that are present in the intestinal tract (Amato, 2013; Cryan et al., 2020). This intricate community has attracted significant attention in recent decades in human clinical research as it plays a crucial role in many physiological and pathological processes such as digestion, metabolism, pathogen defence, and host immunity, finally affecting the behaviour of the host (Di Vincenzo et al., 2024) through the intricate connection that exists via the gut–brain axis (Lima‐Ojeda et al., 2017). Over the last decade, most of the studies on gut microbiome have been conducted on terrestrial animals, including humans (Lima‐Ojeda et al., 2017) and other mammals (Bercik et al., 2011). In fish, research has mostly focused on farmed fish (Rudi et al., 2018; Xing et al., 2013) and the zebrafish, *Danio rerio*, animal model (de Abreu et al., 2019; Green et al., 2024). Although studies on the gut microbiome in marine fish are limited, particularly for wild fish models, some research has been conducted on wild species, such as the common two‐banded seabream, *Diplodus vulgaris* (Ginevra et al., 2024), demonstrating its involvement in several vital functions, including immunity, development, pathogen control, nutrition metabolism, and digestion (Egerton et al., 2018; Ou et al., 2021). However, the conditions in aquaculture and the wild are vastly different, raising the open question of whether the behaviour of wild marine fish is linked to its gut microbiome.

The gut microbiome is composed of two distinct communities: the allochthonous or transient community, comprising free‐living organisms, and the autochthonous or stable community, which adheres to the intestinal mucosa and defines the core microbiome (Egerton et al., 2018; Viver et al., 2023a; Viver et al., 2023b). The core microbiome represents the set of bacteria and related genes that are characteristic of a certain host or environment and may play an essential role in maintaining the host's well‐being (Neu et al., 2021). Additionally, non‐core bacteria may also be relevant, as they have been linked to hormone production and reproductive success in black rhinoceros, *Diceros bicornis* (Antwis et al., 2019). Furthermore, they have been discovered to be responsible for the compositional variation in the gut microbiome following dietary changes. Given that diet is crucial for fitness, it may drive important ecological outcomes. For instance, it has been proposed that the non‐core microbiome plays a significant role in the capacity to colonize new niches in the eastern subterranean termite, *Reticulitermes flavipes* (Benjamino et al., 2018).

Numerous factors can contribute to the variability of gut microbiome in fish. These include the host's genetic background and other factors such as size (Yukgehnaish et al., 2020), diet, trophic level, temperature, salinity (Egerton et al., 2018; Ou et al., 2021), and habitat (Kim et al., 2021; Teng et al., 2022). Regarding space use (e.g. home range, territory size, or daily activity), some studies have found links with the gut microbiome in terrestrial animals. For example, a higher ratio of Firmicutes to Bacteroidetes was found to be associated with the core range area (i.e. the size of the core area within the home range) in two ungulate species, *Oreamnos americanus* and *Odocoileus virginianus* (Wolf et al., 2021). Another study found that gut bacteria transmitted through space‐use overlap among individuals were primarily aerotolerant spore‐forming genera in wild mice, *Apodemus sylvaticus* (Raulo et al., 2024). Something similar occurred in semi‐feral Welsh mountain ponies, *Equus ferus caballus*, where the spatial distribution of individuals was correlated with gut microbiome composition (Antwis et al., 2018). In fish, it was found that the composition and structure of the gut microbiome differ with habitat at a small spatial scale. Specifically, the proportion *of Posidonia oceanica* within the home range was found to be related to the composition of the gut microbiome in *Diplodus vulgaris* (Ginevra et al., 2024). In general, gut microbiome variability with respect to space use in free‐living fish is poorly studied due to the challenges of tracking individuals in the open ocean compared to GPS‐based data obtained from terrestrial animals.

Another relevant aspect to consider is that in group‐living animals, social interactions may play an important role in shaping the microbiome through microbial transmission pathways among members (Raulo et al., 2021; Tung et al., 2015). In many species, intraspecific social interactions are crucial for the ecological and evolutionary processes of wild‐living animal populations (Kurvers et al., 2014; Silk & Fisher, 2017). Better understanding of collective phenomena through the study of social interactions can clarify how species adapt to environmental perturbations and how their interactions shape ecosystem functions (Ings et al., 2009; Tylianakis, 2008). Significant links between animals' social behaviour and microbiomes have been discovered in terrestrial animals in recent years (Johnson et al., 2022; Perofsky et al., 2017; Vernier et al., 2020). While the causal mechanisms remain unclear, it is well established that social interactions can predict the composition and function of the microbiome (Tung et al., 2015). At the same time, the microbiome itself influences host social communication via the gut–brain axis (Mayer et al., 2015), through metabolite production and communication with the central nervous system (Soares et al., 2019).

The term ‘social microbiome’ has been defined as the microbial metacommunity that exists within a group of social animals or a social network (Sarkar et al., 2020). Several studies in humans have found a relationship between the composition of this social gut microbiome and social contact. For example, households have a more similar bacterial community compared to members from other families (Yatsunenko et al., 2012). The relationship between the social network and gut microbiome composition in animals is predominantly documented in mammals, particularly in primates. A study investigating the relationship between the social network and the gut microbiome composition in two social groups of wild baboons in Kenya found that social interactions directly affected gut microbiome composition. Similarly, social group membership was found to have a relationship with individual's microbiome in wild Verreaux's sifakas, *Propithecus verreauxi* (Perofsky et al., 2017). Similar results were found in other mammals, where Raulo et al. (2021) found that individuals with stronger social association had a more similar gut microbiome. Beyond mammals, evidence is emerging that social interactions can also shape the gut microbiome in other animal groups. For instance, in birds, Maraci et al. (2022) showed that the social environment during early development influences gut microbiome composition in zebra finches, *Taeniopygia guttata*. Similarly, in social insects such as honeybees, *Apis mellifera*, close contact and colony organization mediate microbial transmission and gut community structure (Liberti et al., 2022; Vernier et al., 2020). These findings suggest that the interplay between social behaviour and the gut microbiome may represent a widespread phenomenon across the animal kingdom, rather than being restricted to mammals. Despite this growing body of research, studies exploring this relationship in fish are very scarce (Butt & Volkoff, 2019; Singh et al., 2019), and its underlying mechanisms in wild‐living fish populations and communities remain poorly understood (Raulo et al., 2024).

Given the challenges associated with direct animal observation across various species and the constraints inherent in traditional data‐collection methodologies, high‐throughput tracking systems provide a way to monitor movements and acquire precise and simultaneous information from a large number of individuals (Alós et al., 2022; Nathan et al., 2022). The use of miniature animal‐attached electronic tags enables the remote collection of fine‐scale spatial data (required to properly characterize space use) on hundreds of individuals (required to properly quantify social organization) in fluctuating environments (Krause et al., 2013). In marine fish, high‐resolution acoustic telemetry systems allow for locating and obtaining the trajectories of animals tagged with acoustic transmitters (Lennox et al., 2023), thereby facilitating the reconstruction of social networks in wild marine fish populations (Aspillaga, Arlinghaus, Martorell‐Barceló, Follana‐Berná, et al., 2021).

Here, we investigate the correlation between space use, social organization and the gut microbiome in a model marine fish species, the pearly razorfish, *Xyrichtys novacula*, lately being used to characterize social structure at high resolution (Aspillaga, Arlinghaus, Martorell‐Barceló, Follana‐Berná, et al., 2021). This small wrasse (Labridae) inhabits sandy bottoms of coastal warm waters of the Mediterranean Sea and Atlantic Ocean. The species has a carnivorous diet, primarily preying on benthic invertebrates, such as crustaceans and molluscs, although the feeding habits vary with fish size (Castriota et al., 2005). Prey diversity can also vary depending on the habitat, as this species lives on homogeneous sandy bottoms, often adjacent to seagrass meadows or coral reefs, or in mosaics of sand and seagrass meadows of *Cymodocea* sp., *Zostera* sp., and *P. oceanica* (Espino et al., 2015). Several reasons make it an attractive candidate for behavioural and ecological studies, including its burrowing behaviour to rest and avoid predators mainly at night, but also displayed during the day (Alós et al., 2017), its easily measurable daily rhythms (Martorell‐Barceló et al., 2024), its well‐studied social network (Aspillaga, Arlinghaus, Martorell‐Barceló, Follana‐Berná, et al., 2021), and its significant socioeconomic importance in the local fishing sector (Alós et al., 2016). The social structure and distinctive sex‐dimorphic mating behaviour of the pearly razorfish are particularly intriguing. This species is a protogynous hermaphrodite, exhibiting pronounced sexual dimorphism. Males are larger than females and display distinct colour patterns.

The social organization of the pearly razorfish is based on a harem system, where each harem consists of one male and a small group of females (Marconato et al., 1995). Males display agonistic territorial behaviour towards other males (Shen & Clark, 2016). Within these harems, the male territory encompasses several female territories, which are characterized by high residency and limited spatial overlap among harems (Aspillaga, Arlinghaus, Martorell‐Barceló, Follana‐Berná, et al., 2021; Shen & Clark, 2016). Its sedentary nature and small home range (Alós et al., 2012; Shen & Clark, 2016) make it ideal for social network studies in marine environments, as high‐resolution tracking demands a large network of locally spaced receivers and limited movements of the study organism outside the tracking array. Recent high‐resolution tracking revealed high residency of pearly razorfish in its natural coastal environment, with males having an average and relatively small territory of 326 m^2^ (radius = ~10 m) and females 79 m^2^ (radius = ~5 m) (Aspillaga, Arlinghaus, Martorell‐Barceló, Follana‐Berná, et al., 2021). There was an overlap between the territories of males and females of the same harem but not between individuals of the same sex. Such conditions may facilitate the development of a gut microbiome that varies among individuals of different harems. In this work, we hypothesized that habitat use, space use, and the social organization of the tracked individuals affect the composition of the gut microbiome. Specifically, we predicted that individuals living close to resource‐rich habitats (e.g. seagrass meadows) would have a richer gut microbiome community. In addition, we hypothesized that fish from the same harem would exhibit a more similar gut microbiome due to increased social contact and potentially enhanced transmission pathways. The present study serves as a baseline investigation into the correlation between space use, habitat, social organization, and the gut microbiome in wild marine fish.

## 2 MATERIALS AND METHODS

### 2.1 Study site

**FIGURE 1:**
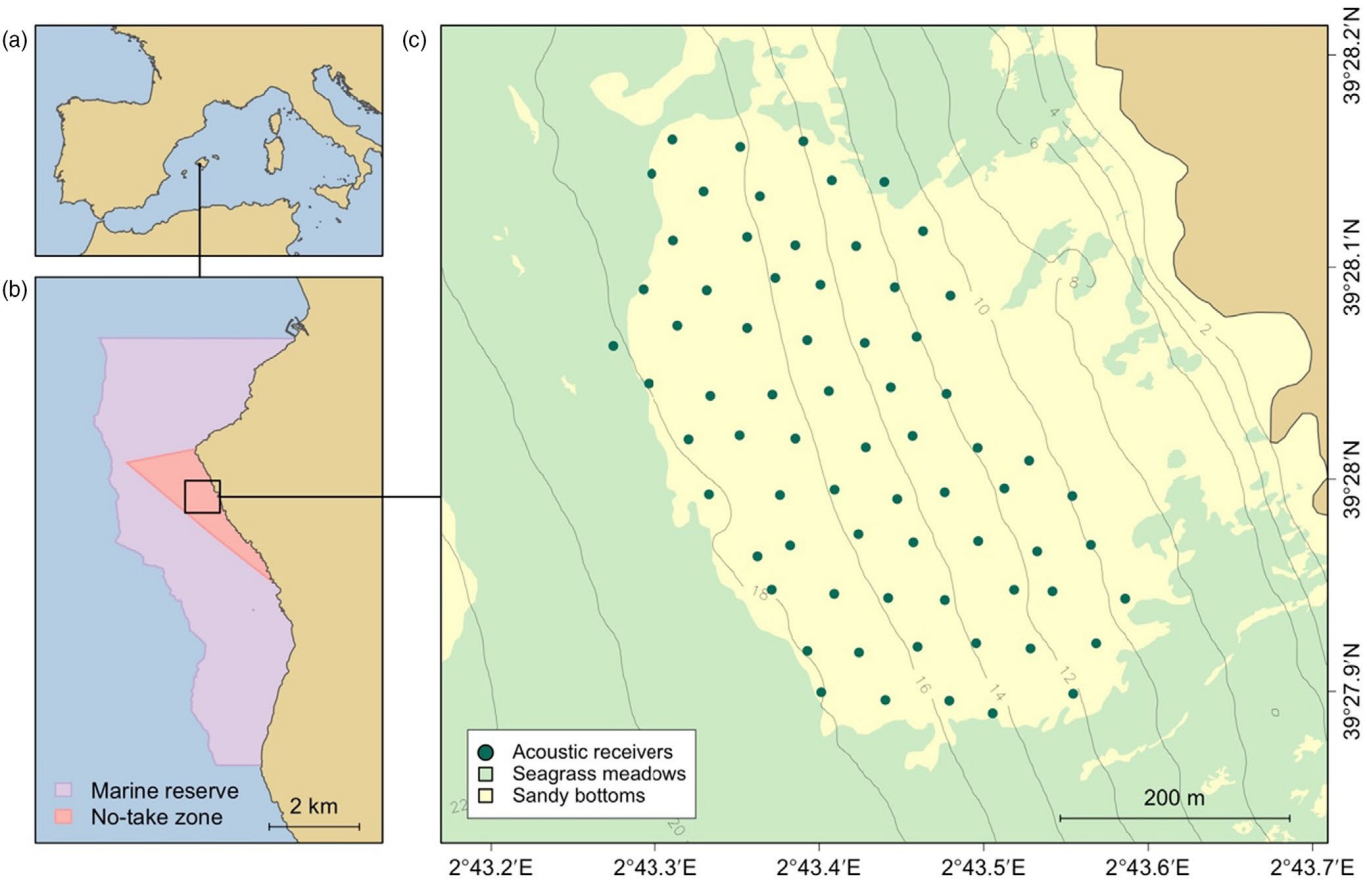
(a, b) Geographic location of the study area within the Marine Reserve of Palma Bay (Mallorca, Balearic Islands, Spain). (c) Deployment of the acoustic telemetry system, showing the position of each receiver (WHS‐4250L) within the high‐resolution tracking experiment area. Note that the area is surrounded by *Posidonia oceanica* meadows (green patches).

The studied area comprised a small sandy patch of 12.5 ha (600 m × 270 m) in the Marine Reserve of Palma Bay, Mallorca, Balearic Islands, Spain (Figure 1). Depths ranged from 12 to 17 m, and the area was surrounded by a *P. oceanica* seagrass meadow, which constrains the habitat of adult pearly razorfish. The area has been thoroughly studied in the past (Aspillaga, Arlinghaus, Martorell‐Barceló, Follana‐Berná, et al., 2021) and has been found to consistently keep a structured resident population of the pearly razorfish over extended periods of time (months). For more details about the study site, see Aspillaga, Arlinghaus, Martorell‐Barceló, Follana‐Berná, et al. (2021).

### 2.2 High‐resolution acoustic tracking experiment

The pearly razorfish individuals were tracked using the Juvenile Salmon Acoustic Telemetry System (JSATS, McMichael et al., 2010) manufactured by Lotek Wireless Inc., Canada. The system was composed of WHS‐4250L receivers and L‐AMT series acoustic transmitters working at 416.7 kHz. Receivers were installed in the study area spaced 50 m apart to ensure overlapping acoustic ranges and allow signal positioning (Figure 1). Transmitters were programmed to emit coded acoustic signals every 2 to 10 s (depending on the model). For more detailed specifications of the tracking system and assessment of high functionality in terms of tracking performance and output of extremely high‐resolution positions with limited error, see Aspillaga, Arlinghaus, Martorell‐Barceló, Barcelo‐Serra, et al. (2021).

The study period spanned from April to October 2019, covering the reproductive season of the species. Initially, a total of 320 individuals (195 females and 125 males) of pearly razorfish were captured in the same area and tagged between April and July 2019. Individuals were captured using experimental rod‐and‐reel gear with live shrimps as bait, sized (total length), and sexed before being tagged. Fish were then anaesthetised by submersion in a solution of 0.1 g^−1^ tricaine methanesulfonate (MS‐222). L‐AMT transmitters were implanted in the peritoneal cavity through a small incision in the ventral side, which was subsequently closed using surgical stitches. Each individual was tagged with a transmitter appropriate to its body size. The specific transmitter model and type used for each individual, based on size, can be found in Table A1, Supporting Information A. Finally, fish were moved to a tank of clean sea water until complete recovery of normal behaviour and released at the same capture location. The entire procedure lasted up to 20 min.

### 2.3 Tracking data processing and positioning

The receiver's acoustic detection data were transferred to a computer using the WHS Host software (Lotek Wireless Inc.). Transmitter positions were estimated from the signals concurrently detected by at least three receivers utilizing a hyperbolic multilateration algorithm through the supplier‐supplied UMAP software (Lotek Wireless Inc.). The estimated raw positions (x and y coordinates) were then imported into R (R Core Team, 2024) for pre‐processing following the methodology outlined in Aspillaga, Arlinghaus, Martorell‐Barceló, Barcelo‐Serra, et al. (2021). First, a sequential position filter was applied to remove spurious estimations: twin positions (duplicated time stamps), positions with a dilution of precision value larger than one (uncertain positions), and positions generating unrealistic spikes in the trajectory (trajectory filter based on step lengths and turning angles) were removed from the dataset. Then, we fit a continuous‐time correlated random walk movement model (CTCRWMM, Johnson et al., 2008), which accounted for the positioning error, to correct the remaining spurious positions and generate regular trajectories at 5‐s timestamps. To ensure that our data adequately represented the behaviour of the pearly razorfish population and their associations, only days with data from more than 50 individuals and individuals with more than 7 tracking days were selected for posterior analyses. After all the data processing and analyses, a position dataset composed of 7930 one‐day‐long trajectories (with 0.2 m error) was obtained. The final dataset comprised 232 pearly razorfish individuals (153 females and 79 males).

### 2.4 Space and habitat use characterization

Trajectories were used to compute a first set of variables describing the space use behaviour of each individual on a daily basis. First, the total daily travelled distance (in m), used as a proxy for fish activity, was calculated as the sum of all the distances between consecutive positions in each tracking day. Second, the daily home range (in m^2^) was estimated as the area of the 95% probability contour of a Kernel Density Estimator (KDE) applied to the daily positions using the MASS package for R (Venables & Ripley, 2002). Finally, to account for the effect of the nearby *P. oceanica* seagrass habitats, we calculated the minimum distance (in m) between the centroid of each daily home range contour and the surrounding seagrass meadow, based on the local maps of marine habitats.

### 2.5 Individual sociability scores and social organization

We constructed the social network of the population based on accurate individual trajectories, generating two groups of variables: an association matrix and individual scores of social interactions. The association matrix described population structure, with associations between two individuals defined based on their positions at 5‐min intervals. If their trajectories were within 5 m, they were considered positively associated, following Aspillaga, Arlinghaus, Martorell‐Barceló, Follana‐Berná, et al. (2021). In this matrix, higher values represented stronger associations, reflecting pairs of individuals that were more frequently detected within this distance threshold. Association weights were calculated by dividing the number of associated intervals by the total intervals for both individuals. We also created matrices for minimum distances of 10 and 20 metres to evaluate the impact of different thresholds (see Supporting Information A, Tables A2–A4). Individual social interaction scores were calculated using the *igraph* package in R (Csardi & Nepusz, 2006). In the social network, each node represented an individual and the edges represented their associations. The degree of a node reflected its connections or associations to other individuals, while strength represented the total weights of the associations linked to that node. Betweenness centrality measured how frequently an individual lay on the shortest paths connecting others, highlighting its role as an intermediary. Lastly, eigenvector centrality measured an individual's overall influence in the network by considering both its connections and the centrality of its neighbours, calculated as the sum of their centralities (Clauset et al., 2004; Farine & Whitehead, 2015; Layeghifard et al., 2017).

### 2.6 Gut microbiome composition: 16S rRNA Gene amplicon diversity

After concluding the tracking period, we recaptured 26 of the tagged individuals using standardized rod‐and‐reel fishing. The total length (TL) of the individuals ranged from 9.6 to 18.8 cm for females and 16.3 to 22.3 cm for males. These sizes fall within the typical adult range of *X. novacula* (Cardinale & Ardizzone, 1998). Therefore, ontogenetic shifts in gut microbiome composition are not expected across this range. The gastrointestinal tracts were collected under aseptic conditions and preserved in RNA later just after capture. For microbiome collection, the portion of the intestine was longitudinally opened, and the inner intestinal walls were carefully scraped using a round‐edge spatula, allowing for the collection of both allochthonous and autochthonous microorganisms. DNA extraction and amplicon‐sequencing analysis were described in Viver et al. (2023a) and Viver et al. (2023b). Briefly, microbial DNA extraction was carried out on 100 μL of mixed gut biomass utilizing the QIAamp DNA Microbiome Kit (Qiagen, Germany). Illumina amplicon‐sequencing was conducted targeting the V3–V4 variable regions of the 16S rRNA gene using bacterial primers Bakt_341F (5′‐CCTACGGGNGGCWGCAG‐3′) and Bakt_805R (5′‐GACTACHVGGGTATCTAATCC‐3′) (Herlemann et al., 2011), which included the forward (5′—TCGTCGGCAGCGTCAGATGTGTATAAGAGACAG—3′) and reverse (5′—GTCTCGTGGGCTCGGAGATGTGTATAAGAGACAG—3′) Illumina sequencing adapters (Illumina, n.d.). Sequencing was executed on an Illumina MiSeq instrument (2 × 250 bp) (FISABIO, Valencia, Spain). Quality assessment was performed using the Qiime2 bioinformatics platform (Bolyen et al., 2019) with parameters ‐‐p‐trunc‐len‐f 280, ‐‐p‐trunc‐len‐r 220, ‐‐p‐trim‐left‐f 19, and ‐‐p‐trim‐left‐r 22. Amplicon sequence variants (ASVs) were obtained using the DADA2 software implemented in Qiime2 (Bolyen et al., 2019). The longest sequence of each ASV was chosen as the representative. The SINA tool (Pruesse et al., 2012) integrated into the ARB software (Ludwig et al., 2004) was employed to align the representative sequences before incorporating them into the SILVA REF138 and LTP_12_2020 (‘The All‐Species Living Tree’ Project) reference databases (Ludwig et al., 2021; Quast et al., 2012; Yarza et al., 2008) using the parsimony tool available in ARB. The closest relatives from both databases (SILVA REF138 and LTP_12_2020) were selected for de novo reconstruction of a neighbour‐joining tree with a 30% conservational filter. Finally, representative ASV partial sequences were inserted using the Parsimony tool in the ARB program package (Ludwig et al., 2004) and clustered into operational phylogenetic units (OPUs) following our standard manual supervision (Mora‐Ruiz et al., 2015). An OPU constitutes the smallest clade containing our sequences affiliated with at least one of the sequences present in public repositories and, when possible, the sequence of one type of strain (Mora‐Ruiz et al., 2015).

### 2.7 Data analysis

We investigated the correlation between the gut microbiome composition and space use, habitat use, and social network structure within our case study population. The analyses included three main approaches: univariate analysis of diversity indices (General Linear Models), multivariate analysis of gut microbiome composition (distance‐based redundancy analysis [dbRDA]), and an examination of the correlation between the social network association matrix and the gut microbiome composition matrix (Mantel test). While individual scores from the social network, used in both univariate and multivariate analyses, focused on the entire social network (comprising 232 tracked individuals), Mantel test analysis focused on associations between the recaptured individuals and their gut microbiome data. Each approach was performed separately for the core microbiome and the non‐core microbiome. To define these groups, we constructed a presence‐absence data matrix to determine the occurrence of every OPU in each individual. The core gut microbiome was defined as OPUs present in at least 80% of the individuals, following the criteria established by Neu et al. (2021). The non‐core microbiome included OPUs found in at least 10% but fewer than 80% of individuals, thereby excluding core gut microbiome members.

The OPU analysis of the samples included in this study was previously described in Viver et al. (2023a) and Viver et al. (2023b). Given that all samples were sequenced in the same Illumina run and exhibited a comparable number of reads, we used the non‐normalized dataset. Diversity indices and multivariate analyses were computed from relative‐abundance matrices, which inherently account for differences in sequencing depth across samples. Bray–Curtis dissimilarities (for PCoA) and distance‐based RDA (dbRDA) were also calculated using these relative‐abundance data. All analyses described in this section were conducted in R version 4.3.3 (R Core Team, 2024). Detailed descriptions and taxonomic information of the OPUs are provided in the Supplementary Material of Viver et al. (2023a) and Viver et al. (2023b).

#### 2.7.1 Univariate analysis of diversity indices in the gut microbiome

We first computed three diversity indices related to the OPUs in the gut for each individual using the Vegan Package (Oksanen et al., 2012): richness (i.e. number of species or OPUs), Shannon Index (i.e. diversity index that measures diversity by considering both species richness and evenness, being more sensitive to rare species), and Simpson Index (i.e. diversity index that reflects dominance within the community as it is more influenced by the most abundant species). The combined use of the indices provides a more comprehensive assessment of microbial community diversity. We modelled each diversity index, calculated for the core and non‐core microbiome, against the explanatory variables generated from the tracking data: territory size, activity, distance to nearest seagrass patch, degree, strength, betweenness centrality, and eigenvector. In addition, we considered the size of the fish as a co‐variate in the models to control for the effect of size and sex‐related differences (as females are smaller than males). Due to the large number of variables, and to avoid co‐linearity issues, we explored the correlation among them using the *corrplot* package (Taiyun Wei et al., 2022). For each pair of variables found to be highly correlated, one variable was selected for the subsequent analysis. The final selected variables were body size (correlated with being male), territory size (correlated with activity), degree (correlated with betweenness centrality), strength (correlated with eigenvector centrality), and distance to *P. oceanica* seagrass meadow. In addition, after fitting a GLM for each diversity index, we applied the *stepAIC* function to select the significant variables using the AIC criterion in both directions (Venables & Ripley, 2002). When performing the GLM using the richness as the response variable, a Poisson distribution with a log link function was used since the data were counts. In the other cases, we considered a normal distribution of the data. All variables were scaled, as they originally displayed a wide range of different units.

#### 2.7.2 Multivariate analysis of gut microbiome composition matrix

While univariate analysis focused on single variables, multivariate analysis examined how the composition and community‐level dynamics (i.e. the abundance matrix of OPUs) of the gut microbiome correlated with a set of explanatory variables, including body size, territory size, degree, strength, distance to the seagrass meadow, and sex. To quantify differences in microbiome composition between individuals, we computed a Bray–Curtis dissimilarity matrix using the *vegdist* function in the Vegan package (Oksanen et al., 2012), where higher values indicate greater dissimilarity and lower values more similar microbial communities.

We then applied a Principal Coordinate Analysis (PCoA) to visualize general variability patterns in microbiome composition using the *cmdscale*() function, which is included in base R. Subsequently, a distance‐based redundancy analysis (dbRDA) based on the Bray–Curtis dissimilarity index was conducted, followed by ANOVA, to test the relationship between the explanatory variables (mentioned above) and the composition matrix of both the core and non‐core gut microbiome. In parallel, a PERMANOVA was performed using the *adonis2*() function from the Vegan package in R to evaluate the effect of the categorical variable sex on the microbiome composition. Finally, we tested for differences in within‐group variability (dispersion) between sexes using *betadisper*() (Vegan package) on Bray–Curtis dissimilarities, followed by ANOVA and a permutation test (9999 permutations).

#### 2.7.3 Correlation between the social association matrix and the gut microbiome composition matrix

The last analysis examined the correlation between the social association matrix and the gut microbiome composition matrix. The objective was to assess whether social structure was associated with variation in gut microbiome composition. To test this, we used two matrices. The first was the social association matrix, derived from the Social Network Analysis, in which associations were defined as interactions between two individuals within a minimum distance of 5 m. The second was the gut microbiome dissimilarity matrix, represented by the Bray–Curtis dissimilarity matrix generated in the previous analysis. We then compared these two matrices using a Mantel test with Spearman's rank correlation, applying a null model with 9999 permutations to evaluate whether variation in social association strength was related to differences in gut microbiome composition. Additionally, the social association matrix was recalculated using minimum distances of 10 and 20 m between individuals to assess whether the observed patterns were consistent across spatial scales.

## 3 RESULTS

### 3.1 Social network structure and recaptures

**FIGURE 2:**
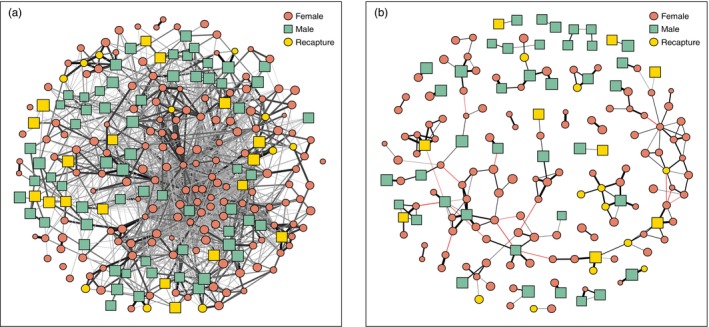
Social network of the pearly razorfish population for the entire tracking period showing associations (connections) at a threshold of a minimum distance of 5 m. Recaptured individuals (*N* = 26) used in the present study are indicated in yellow. Node size is proportional to the body size of each individual. Darker and thicker edges represent higher edge weights, indicating more frequent social contact between individuals. The shaded clusters indicate the different groups identified by the fast‐greedy modularity optimization algorithm for community structure detection (Clauset et al., 2004). Red edges indicate associations between individuals from different groups. (a) Entire social network for all the 232 pearly razorfish individuals. (b) Subset of the social network displaying the strongest 10% edges (i.e. the strongest social connections among individuals).

The reconstruction of the social network revealed a harem‐like social structure (Figure 2). The pearly razorfish exhibits a hierarchical social organization composed of harems. Within each harem, one male typically maintains a territory that encompasses the territories of three to four females, as described in detail earlier in this study and in the methodology and technical specifications provided by Aspillaga, Arlinghaus, Martorell‐Barceló, Barcelo‐Serra, et al. (2021). Of the 232 tagged pearly razorfish individuals forming the social network, 26 were randomly recaptured (indicated as ‘recaptures’ in Figure 2), including 18 males and 8 females. The bacterial 16S rRNA gene from their gut contents was sequenced to assess microbiome diversity and composition.

### 3.2 Gut microbiome composition

The diversity of the intestinal microbiome of the 26 individuals of pearly razorfish was analysed using 16S rRNA gene amplicon sequencing. A total of 847,231 high‐quality 16S rRNA gene sequences were clustered in 1019 OPUs, primarily attributed to the phyla Proteobacteria (*n* = 472), Firmicutes (*n* = 200), Actinobacteria (*n* = 111), Bacteroidetes (*n* = 70), and Planctomycetes (*n* = 33), accounting for 87% of the gut microbiome (Viver et al., 2023a, 2023b). The most abundant OPU was *Ralstonia* sp. (OPU 0522), with an average relative abundance of 16 ± 23.4%. The second most abundant OPU was *Paeniclostridium* sp. (OPU 0315), with an average abundance of 3.7 ± 13.4% of the total OPUs detected. Cyanobacteria (OPU 1060) and chloroplasts (OPU 1059) sequences were identified in all individuals, with average relative abundances of 7.2 ± 6.9% and 3.5 ± 2.3%, respectively. *Ruegeria* sp. (OPU 0816), with an average relative abundance of 1.5 ± 1.3%, along with *Ralstonia* sp., were the only OPUs found in all individuals of the study (Supporting Information B, Table B1).

**FIGURE 3:**
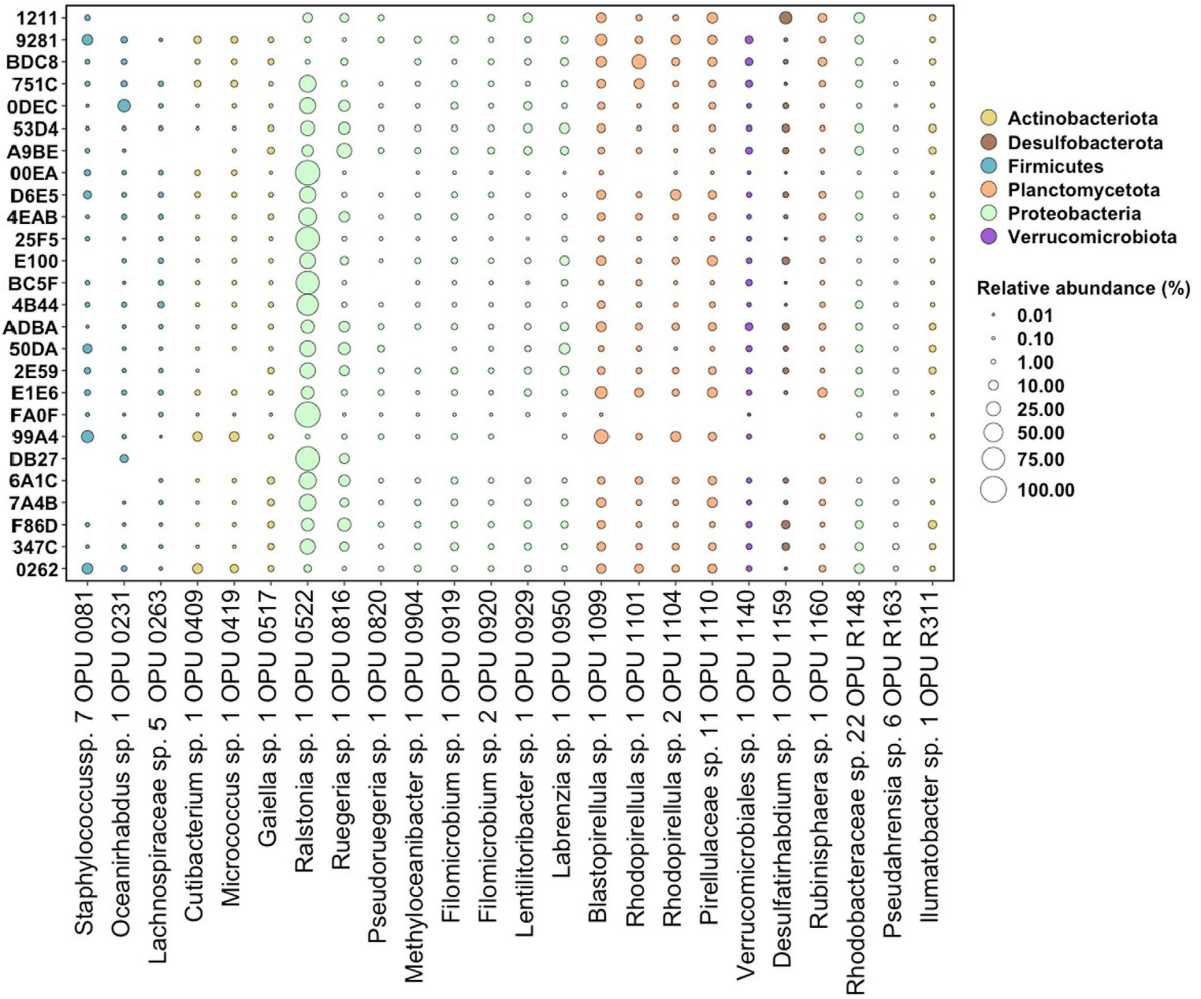
Relative abundances (%) of the OPUs present in >80% (core microbiome; *x*‐axis) of the studied individuals (*y*‐axis), marked with different colours according to their major phyla: Yellow (Actinobacteriota), brown (Desulfobacterota), blue (Firmicutes), orange (Planctomycetota), green (Proteobacteria), and purple (Verrucomicrobiota). The y‐axis shows the ID of the individuals recaptured after the tracking period.

The core microbiome was defined based on the OPUs present in >80% of the individuals, with a total of 24 OPUs (Supporting Information B, Table B2). Six phyla formed the core microbiome, with the phylum Proteobacteria representing 42% of the core microbiome, followed by the phylum Planctomycetota (21%), Actinobacteriota (17%), Firmicutes (12%), and, in smaller proportions, Verrucomicrobiota (4%) and Desulfobacterota (4%). The relative average abundance was quantified (4.2 ± 7.2% OPU average abundance per sample), with the most abundant OPU of the core gut microbiome being *Ralstonia* sp. (OPU 0522), with an average abundance of 37.2%. The second most abundant OPU of the core was *Ruegeria* sp. (OPU 0242), with an 7.7% average abundance. Moreover, the two most recurrent OPUs per individual were *Ralstonia* sp. and *Ruegeria* sp., present in all fish (Figure 3, Supporting Information B, Table B2).

### 3.3 Univariate analysis of diversity indices in the gut microbiome

The analysis of correlation among explanatory variables (see the correlation matrix in Supporting Information C, Table C1) retained five variables to be fitted in the GLMs: body size, territory size (positively correlated with activity), distance to the nearest seagrass patch, degree (positively correlated with betweenness centrality), and strength (positively correlated with eigen centrality). No significant correlations were found between OPUs richness, Shannon and Simpson diversity indices of the core microbiome, and all five independent variables tested (GLM, *p*‐value > 0.05, Supporting Information C, Table C1). This result suggests that the core microbiome was relatively stable and not heavily influenced by the tested variables. Results were different when considering the non‐core microbiome, where significant correlations were found in the GLM (Supporting Information C, Table C2) between the number of OPUs and body size (*p*‐value = 0.026, estimate = 0.06), and distance to *P. oceanica* (*p*‐value < 0.001, estimate = −0.11). The significant positive relationship between the number of OPUs and body size suggested that larger fish harboured a greater number of non‐core OPUs. Regarding the distance to the nearest patch of seagrass of *P. oceanica*, the results suggested a negative significant effect on the number of non‐core OPUs, meaning that fish with the territory located closer to the seagrass meadow had a higher number of OPUs. Although the number of bacterial species is suggested to vary with certain variables in the non‐core microbiome, the diversity (Shannon Index) and dominance (Simpson Index) of species within the microbiome did not change significantly with the five independent variables (*p*‐value > 0.05 in both cases, Supporting Information C, Table C2).

### 3.4 Multivariate analysis of gut microbiome composition matrix

**FIGURE 4:**
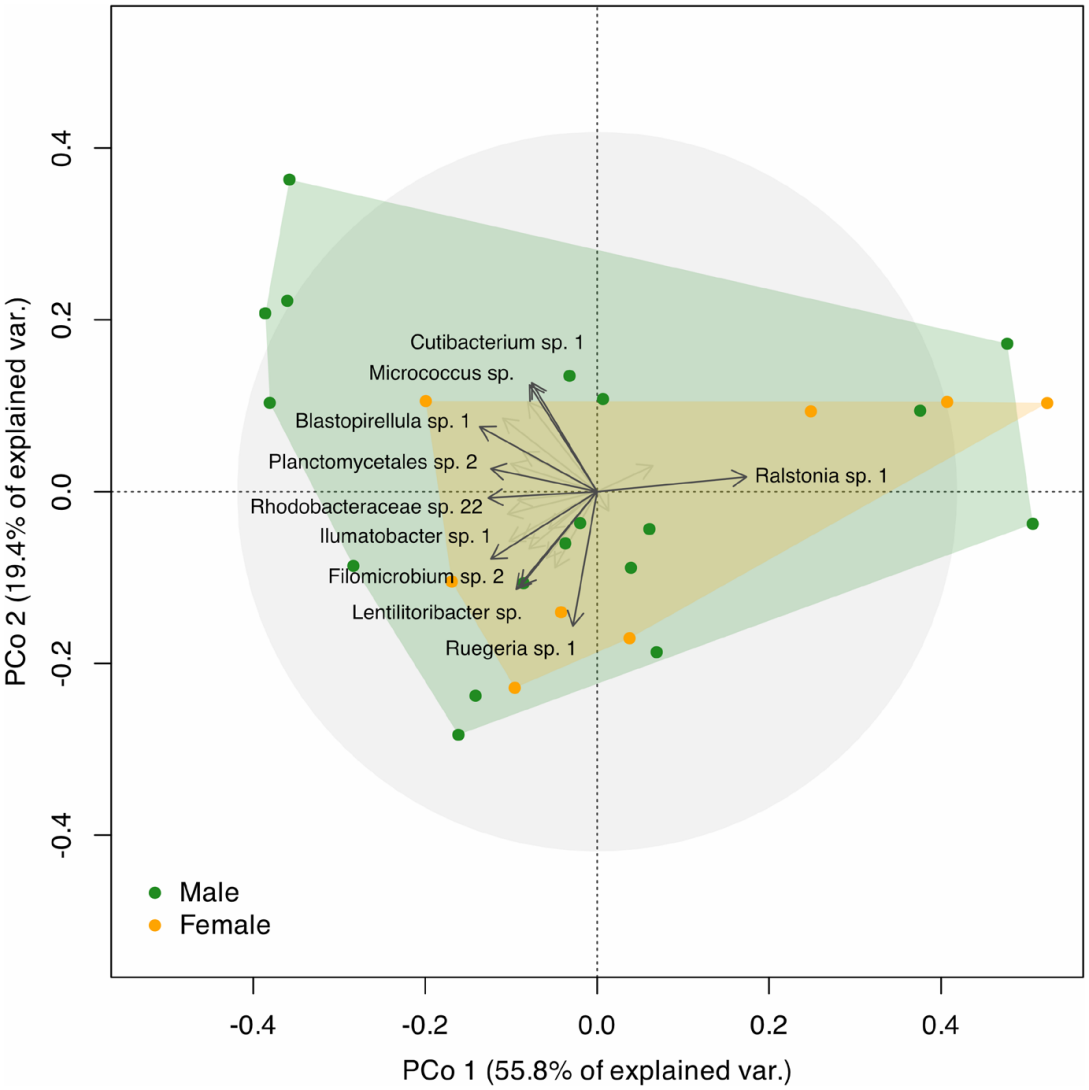
Principal Coordinate Analysis (PCoA) of the core gut microbiome of all individuals grouped by sex. Males (green) showed greater dispersion in microbiome composition compared to females (orange) in the ordination space. Each point represents an individual, and each arrow represents an OPU with its contribution to the variability. OPUs that contribute most to the variability are named in black.

**FIGURE 5:**
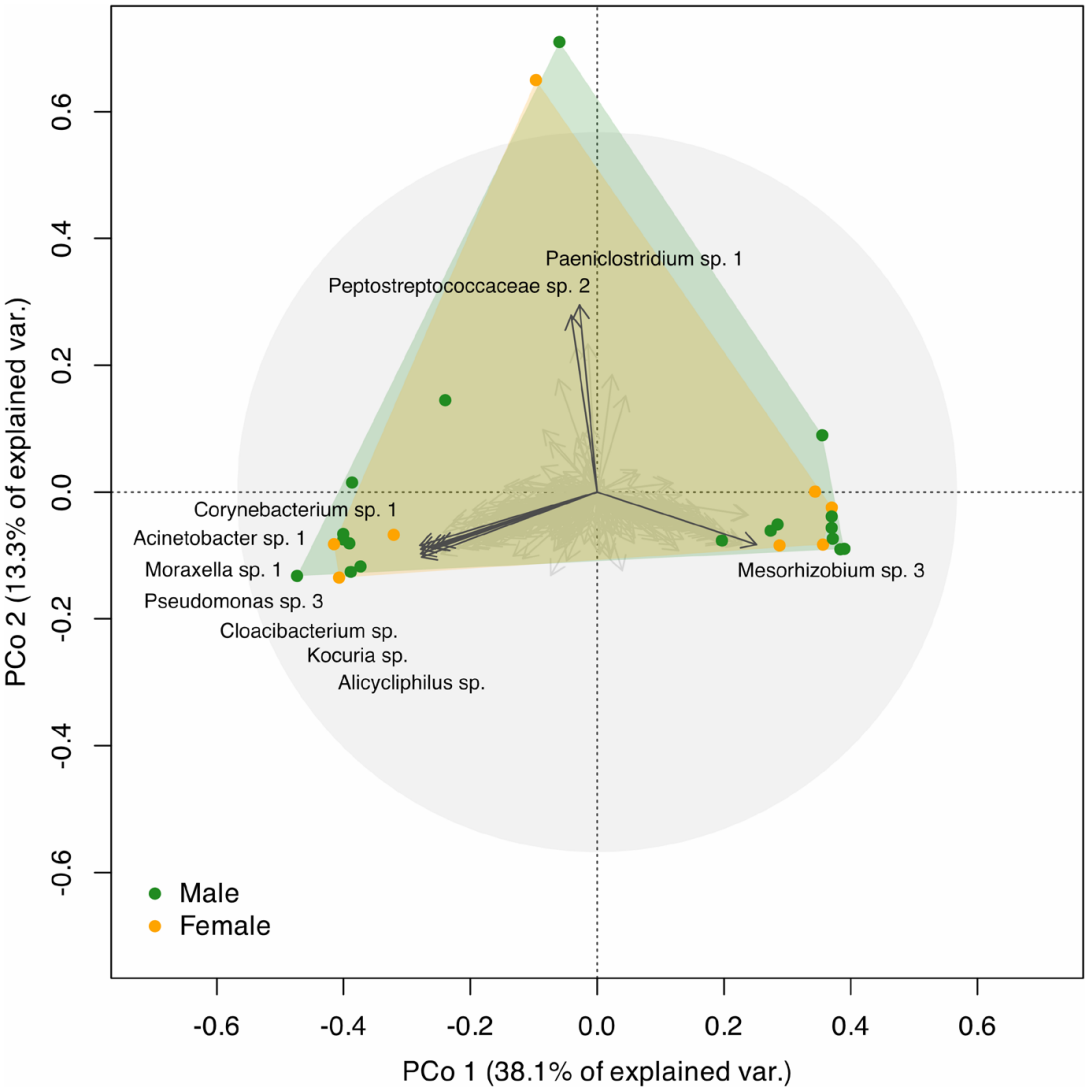
Principal Coordinate Analysis (PCoA) of the non‐core gut microbiome of all individuals grouped by sex. Males (green) and females (orange) showed minimal variability in microbiome composition, with no clear differences between sexes in the ordination space. Each point represents an individual, and each arrow represents an OPU with its contribution to the variability. OPUs that contribute most to the variability are named in black.

Multivariate analyses were performed to investigate the relationship between the composition of the gut microbiome and the sex of the pearly razorfish. First, exploratory analyses were conducted using principal coordinate analyses (PCoAs) for the core (Figure 4) and the non‐core gut microbiome (Figure 5). In the core microbiome, males showed greater dispersion in microbiome composition compared to females in the ordination space (Figure 4), and certain bacteria such as *Ralstonia* sp. 1 (OPU 0522), *Ruegeria* sp. 1 (OPU 0816), and *Blastopirellula* sp. 1 (OPU 1099) appeared to contribute to this variability (Figure 4). This pattern was not statistically supported by the beta‐dispersion analysis, which revealed no significant difference in within‐group variability between males and females (mean ± SD = 0.333 ± 0.158 vs. 0.300 ± 0.129; ANOVA, *p*‐value > 0.05). Similarly, in the non‐core microbiome, both sexes displayed minimal variability in composition (0.549 ± 0.070 vs. 0.548 ± 0.098; ANOVA, *p*‐value > 0.05), consistent with the pattern observed in the PCoA (Figure 5). Three distinct bacterial groups were identified: the first included *Paeniclostridium* sp. 1 (OPU 0315) and *Peptostreptococcaceae* sp. 2 (OPU 0314), the second, *Mesorhizobium* sp. 3 (OPU R157), and the third was more diverse, comprising species such as *Kocuria* sp. 1 (OPU 0430) and *Acinetobacter* sp. 1 (OPU 0701) among others (Figure 5). The results of the dbRDA did not reveal significant differences between the composition of the core microbiome and the variables body size (that can be interpreted also as sex differences), degree, strength, territory size, and distance to *P. oceanica* (*p* > 0.05 in all cases; Supporting Information C, Table C3). The analysis of the non‐core microbiome also did not reveal significant differences between the composition of the microbiome and the variables tested (*p* > 0.05 in all cases, Supporting Information C, Table C4). PERMANOVA analysis did not reveal statistically significant differences in the composition of either the core or non‐core gut microbiome between male and female individuals (*p* > 0.05 in both cases; Supporting Information C, Table C4).

### 3.5 Correlation between the association matrix and the gut composition matrix

Mantel tests were performed to investigate the correlation between social associations and the Bray–Curtis dissimilarity matrix of OPUs in the core and non‐core microbiomes. For the core microbiome, a weak but statistically significant correlation was identified at threshold distances of 5 m (Mantel's *r* = 0.125, *p* = 0.009) and 10 m (Mantel's *r* = 0.134, *p* = 0.017; Supporting Information C, Table C5). Given that the social association matrix reflects interaction strength and the Bray–Curtis matrix reflects dissimilarity, these positive correlations indicate that individuals with stronger social associations tended to have slightly more dissimilar microbiomes. However, the effect size was small, indicating a weak relationship. No significant correlation was found at a threshold distance of 20 m (*r* = 0.078, *p* > 0.05; Supporting Information C, Table C5). For the non‐core microbiome, no significant associations were detected at any of the tested distances (Supporting Information C, Table C5).

## 4 DISCUSSION

In this work, we have taken advantage of one of the most detailed datasets of space use in a marine fish species, generated by high‐throughput tracking (Aspillaga, Arlinghaus, Martorell‐Barceló, Follana‐Berná, et al., 2021), to provide the first insights into the relationship between free‐living behaviour, habitat, social networks, and gut microbiome composition in a marine fish. In the univariate analysis, we provide evidence that the body size of fish (in this species also interpreted as sex differences) and the distance to rich biodiversity habitats, such as seagrasses, are correlated with microbial diversity indices (based on OPUs) of the non‐core microbiome. This suggests that the gut microbiome in fish is shaped by a complex array of factors and highlights the importance of distinguishing between core and non‐core microbial communities in microbiome studies, as their nature may differ significantly—an aspect that could be key for future research linking microbiomes and behaviour. In the multivariate analysis, males appeared to show greater variability in their core microbiome composition than females, although this pattern was not statistically supported by the beta‐dispersion test. No differences in overall composition were detected by PERMANOVA. This suggests that sex‐related differences in gut microbiome structure may reflect subtle or context‐dependent effects, influencing individual variability rather than producing distinct compositional profiles. The Mantel test revealed a weak association between social harem structure and microbiome composition, indicating a limited role of social interactions in shaping gut microbiomes at the resolution of our study. Despite this modest effect, our observational approach provides a novel framework to link individual behaviour with microbiome variation in wild fish. By integrating high‐resolution behavioural data with microbiome profiling, this work opens new avenues to investigate the emergence and ecological relevance of a ‘social microbiome’ in marine systems and provides some of the first evidence connecting behaviour and gut microbiota in free‐living fish.

Although there is a wide variety of microorganisms in the intestine, the majority of studies have focused on bacteria because they are the most common group in the fish gut (Rombout Jan et al., 2011). The taxa identified in this study resembled those found in other fish species (Egerton et al., 2018; Roeselers et al., 2011), with Proteobacteria being the dominant phylum. To ensure comparability with these previous studies and achieve a high level of taxonomic precision, our microbiome samples followed a strict, standardized protocol, and the analysis used allowed us to designate each OPU as a different species, maximizing the taxonomic resolution (Mora‐Ruiz et al., 2016).

All the fish in the study shared two bacterial species that could have important functions. The first, *Ralstonia* sp. (OPU 0522), which was the most abundant species in our study, is closely related to *R. mannitolilytica* (GenBank accession AJ270258), showing >98% 16S rRNA gene sequence identity with this species, supporting its taxonomic assignment. This genus is frequently mentioned in the scientific literature since it has been mostly documented as an opportunistic pathogen in humans (Vats et al., 2022). Fish gut microbiome investigations have identified the genus as one of the most prevalent in the gut microbiome community in various fish species (Nayak, 2010), including cultured sea bass, *Dicentrarchus labrax* (Carda‐Diéguez et al., 2014). It has also been identified as a pathogenic microorganism in *Paralichthys olivaceus* (Kim et al., 2023). However, it is also linked to antibacterial activity, biosynthesis of bioactive compounds, and the production of secondary metabolites that are beneficial to the host (Cerezo‐Ortega et al., 2021), suggesting that it may be advantageous to the pearly razorfish and may perform important functions. The second, *Ruegeria* sp. (OPU 0816), was present in all individuals but at lower abundance. It is closely related to *R. lacuscaerulensis* (GenBank accession U77644) and shows >98% 16S rRNA gene sequence identity, supporting its taxonomic assignment. This genus has been proposed to exhibit antimicrobial (Porsby et al., 2008) and probiotic activity in fish (Sonnenschein et al., 2017). These findings suggest that both bacteria, which form part of the core gut microbiome in *X. novacula*, might have important functions. Given the existing research on these two bacterial species found in fish and their reported beneficial functions, they could play various important roles—despite not having been studied in detail—in the fitness, health, and survival of marine fish.

We provide evidence that the non‐core gut microbiome of the pearly razorfish may be shaped by factors such as body size and habitat. Our univariate analysis revealed that the gut of larger individuals, which are males because of the protogynous nature of the species, hosted a greater number of OPUs. This correlation has also been observed in other taxa. For example, in the bee *Tetragonula carbonaria*, gut bacterial richness was associated with larger body size (Liu et al., 2023). A possible explanation for this positive relationship could be that a larger body size increases the available space in the gut to harbour a greater number of bacteria, or it could also increase transit time (Reese & Dunn, 2018). Numerous studies have documented the importance of habitat in defining the gut microbiome (Kim et al., 2021; Teng et al., 2022). In our study, a greater number of bacterial species forming part of the non‐core gut microbiome was observed in fish living near the *P. oceanica* meadow. The higher biodiversity typical of *P. oceanica* habitats (Mari et al., 2021) likely provides a wider range of potential prey, such as crustaceans and molluscs (Como et al., 2008). This abundance of prey likely influences the diets of fish within these habitats compared to those living farther away. These dietary differences could explain the variations observed in the gut microbiome. In contrast to the results observed for the non‐core gut microbiome, body size and habitat did not seem to be correlated with the core microbiome richness (i.e. number of OPUs). Furthermore, individuals' sociability scores (degree and strength) were not related to core microbiome diversity, suggesting that the core microbiome was relatively stable and not strongly influenced by these variables. These findings are highly relevant, as they align with previous studies in humans, where a stable core of bacterial species has been identified alongside a more variable fraction that fluctuates over time in response to external conditions (i.e. the non‐core microbiome; Turnbaugh et al., 2009). Similar patterns have also been reported in animals, including fish, where studies suggest that the non‐core portion is more susceptible to temporal changes and may respond rapidly to shifts in external conditions or resource availability (Sullam et al., 2012). This has important implications, for example, in the aquaculture industry—particularly concerning the use of antibiotics—as the non‐core community may be the most affected.

Some studies suggest that not only do the number of bacterial species and gut microbiome diversity (species richness) affect the host, but also that the composition (relative abundance of the OPUs) has a significant impact on the host (Valdes et al., 2018). Although the dbRDAs did not reveal significant patterns between the composition of either the core or non‐core gut microbiome and the tested variables (size, degree, strength, territory size, and distance to *P. oceanica*), and no significant correlation was found with sex, a visual pattern of greater variability in the core gut microbiome composition of males compared to females was observed in the PCoA. However, this trend was not statistically supported by the beta‐dispersion analysis. This apparent trend could be explained by several factors. First, males have larger body sizes and territories, which could influence gut microbiome variability, allowing for a wider range of bacterial acquisition from different sources and habitats. Similar sex‐dependent patterns in gut microbiome have also been reported in fish, such as freshwater sticklebacks, *Gasterosteus aculeatus* (Bolnick et al., 2014), and in other vertebrates, such as birds, where Corl et al. (2020) found that gut bacterial composition differed by sex in owls, with greater diversity observed in females, which is reasonable, considering that females in these species are larger than males. Second, such sex‐related differences could also be linked to social hierarchies. In this species, males are likely dominant over females and may feed earlier or access richer food resources, which could promote higher gut microbiome diversity and variability. Similar patterns have been reported in other fish species, such as the African cichlid, *Astatotilapia burtoni*, where dominant individuals showed greater gut microbiome diversity, likely due to dietary advantages (Singh et al., 2019).

In the case of the non‐core microbiome, gut microbiome composition showed minimal variability between female and male individuals, and we found three distinct groups of bacteria, mainly clustered into two groups of fish based on the composition of their gut bacterial communities. These clusters were not explained by any of the tested variables and were characterized by specific OPUs. This may suggest the existence of two predominant microbial profiles among the studied fish, while other profiles—such as the group located in the upper part of the plot (Figure 5)—appeared to be less common. The first group was characterized by *Mesorhizobium* sp. 3 (OPU R157). This genus is known for its nitrogen‐fixing capabilities and its presence in marine polychaetes (Summers et al., 2013), suggesting that some individuals might preferentially prey on these organisms. However, it has also been identified as a pathogenic genus in fish (Yang et al., 2022). The OPU identified in this study is phylogenetically close to *M. sediminum*, a species isolated from deep‐sea marine sediments (Yuan et al., 2016) that has not been previously reported in animals. Further research on this specific species is needed to evaluate its potential role in behaviour and health in marine fish. The second group, more diverse in terms of species richness, included taxa such as *Kocuria* sp. 1 (OPU 0430) and *Acinetobacter* sp. 1 (OPU 0701). The former genus has been associated with gut health in fish, specifically with protective roles against crowding‐induced stress, probiotic functions, healthy metabolism, and overall improved health status in teleosts (Li et al., 2025). The genus *Acinetobacter* is commonly found as part of the gut microbiome (Sharma et al., 2024) and has been described as important for metabolism and health across different life stages in fish (Zhou et al., 2025). This suggests that bacteria present in the non‐core microbiome may play significant roles in shaping sex‐related patterns in gut microbiome composition. They could also be involved in various physiological processes, with some potentially associated with disease, while others may provide beneficial functions that are crucial for normal behaviour and the overall health of the pearly razorfish.

We provide a novel framework to investigate the potential role of social structure in shaping the gut microbiome of the pearly razorfish. While social interactions in fishes are generally less evident than in mammals (Raulo et al., 2021), the pearly razorfish species exhibits a well‐defined social organization, with stable harems and frequent interactions within and among groups (Aspillaga, Arlinghaus, Martorell‐Barceló, Follana‐Berná, et al., 2021). Despite this, we found no relationship between OPU diversity and individual sociability metrics (degree and strength), and only a very weak Mantel correlation between social associations and microbiome composition. The significance detected at short spatial thresholds (5–10 m) likely reflects the scale at which interactions and shared environmental conditions occur, rather than direct microbial transmission. In this context, spatial proximity, habitat use, and diet may provide more parsimonious explanations for the observed patterns. Accordingly, our results do not support strong socially mediated homogenization of the microbiome, but instead point to a weak and potentially indirect association between social structure and microbiome variation. This limited role of social interactions is consistent with emerging evidence that fish gut microbiomes are relatively low in diversity and may stabilize early in life, potentially constraining the influence of social transmission compared to other taxa (Limborg et al., 2023).

A relevant methodological aspect of our work is that sociability metrics were derived from a subset of tagged individuals, and the presence of untagged fish may bias estimates of social connectivity (Aspillaga, Arlinghaus, Martorell‐Barceló, Barcelo‐Serra, et al., 2021). While our matrix‐based approach mitigates this limitation by focusing on pairwise associations among sampled individuals, the relatively small sample size (*n* = 26 recaptures) remains a constraint. Future studies including a larger fraction of the population will be essential to robustly assess the role of social processes in shaping microbiome variation. Nevertheless, by integrating high‐resolution tracking data with microbiome profiling, our approach provides a new avenue to explore links between behaviour and microbiome variation in wild fish. This framework opens the possibility to investigate the emergence and ecological relevance of a “social microbiome” in marine systems under natural conditions.

In conclusion, our findings highlight a relationship between space use, habitat, and gut microbiome variation in wild pearly razorfish. The mechanisms underlying these patterns remain uncertain and are likely driven primarily by environmental factors such as habitat use and diet, with at most a limited and indirect contribution of social interactions. Future studies should increase sample size, explicitly control for environmental and dietary effects, and explore temporal dynamics across populations to disentangle these processes (Perofsky et al., 2017). By integrating high‐resolution behavioural tracking with microbiome profiling in a wild marine fish, this study provides a novel framework to investigate links between behaviour and microbiome variation under natural conditions. While evidence for socially mediated microbiome structuring was weak, this approach opens new avenues to test the emergence and ecological relevance of a ‘social microbiome’ in marine systems, and its potential implications for individual performance, social organization and ecosystem functioning.

## AUTHOR CONTRIBUTIONS

Aina Pons, Ignacio A. Catalán, Tomeu Viver, and Josep Alós conceived the ideas and designed the study. Eneko Aspillaga and Josep Alós designed the acoustic telemetry experiment. Robert Arlinghaus provided the necessary material to conduct the acoustic telemetry experiment. Eneko Aspillaga, Martina Martorell‐Barceló, Margarida Barcelo‐Serra, and Josep Alós captured and tagged the fish. Aina Pons and Tomeu Viver performed the molecular laboratory analyses. Aina Pons, Tomeu Viver, Eneko Aspillaga, and Josep Alós conducted the statistical analyses. Aina Pons created the tables and figures and wrote the first draft of the manuscript. All authors contributed critically to the drafts and gave final approval for publication.

## CONFLICT OF INTEREST STATEMENT

The authors declare no conflicts of interest.

## ETHICS STATEMENT

The tagging procedure adhered to the directives outlined in the Spanish Government's regulations (RD 53/2013) and received approval from the University of the Balearic Islands' Committee on the Ethics of Animal Experimentation (Ref. CEEA 107/01/19). Authorization for fishing, handling, and releasing the animals in the Marine Reserve was granted by the Department of Environment, Agriculture, and Fisheries of the Government of the Balearic Islands.

## STATEMENT ON INCLUSION

Our study brought together authors from multiple countries, including scientists based in the region where the research was conducted. All authors were involved from the outset in the study design and later in the revision of the manuscript at various stages, ensuring that the diverse perspectives they represent were considered from the beginning. Both literature published by local scientists and relevant work conducted in other parts of the world were cited.

## Supporting information


**Table A1.** Biometric data of all individuals.
**Table A2.** Associations found between individuals at a distance of 5 m.
**Table A3.** Associations found between individuals at a distance of 10 m.
**Table A4.** Associations found between individuals at a distance of 20 m.
**Table A5.** Social network measures of all individuals at 5‐min intervals at a minimum distance of 5 m.
**Table A6.** All measures required for the calculation of space and habitat use characterization: Activity, home range, and distance to *P. oceanica*.

**Table B1.** Relative abundances of all OPUs in the gut microbiome of the pearly razorfish, followed by the OPU number, the genus of the species, and the species number of the genus. The table also presents the occurrence and the average abundance of each OPU in every individual.
**Table B2.** Relative abundances of the core members in the gut microbiome of the pearly razorfish.

**Table C1.** Results of the univariate analysis of diversity indices in the core gut microbiome of the pearly razorfish. The variables degree and strength were considered with associations at a 5 m distance.
**Table C2.** Results of the univariate analysis of diversity indices in the non‐core gut microbiome of the pearly razorfish. The variables degree and strength were considered with associations at a 5 m distance.
**Table C3.** Results of the multivariate analyses in the core gut microbiome of the pearly razorfish. The variables degree and strength were considered with associations at a 5 m distance.
**Table C4.** Results of the multivariate analyses in the non‐core gut microbiome of the pearly razorfish. The variables degree and strength were considered with associations at a 5 m distance.
**Table C5.** Results of the association matrix and the gut composition matrix (Mantel test) for the core and non‐core gut microbiome of the pearly razorfish at association distances of 5, 10 and 20 m.

## Data Availability

Data available from the Dryad Digital Repository https://doi.org/10.5061/dryad.vdncjsz9x (Alós et al., 2026).
